# The Reproducibility Movement in Psychology: Does Researcher Gender Affect How People Perceive Scientists With a Failed Replication?

**DOI:** 10.3389/fpsyg.2022.823147

**Published:** 2022-06-13

**Authors:** Leslie Ashburn-Nardo, Corinne A. Moss-Racusin, Jessi L. Smith, Christina M. Sanzari, Theresa K. Vescio, Peter Glick

**Affiliations:** ^1^Department of Psychology, Indiana University – Purdue University Indianapolis, Indianapolis, IN, United States; ^2^Department of Psychology, Skidmore College, Saratoga Springs, NY, United States; ^3^Department of Psychology, University of Colorado Colorado Springs, Colorado Springs, CO, United States; ^4^Department of Psychology, University at Albany, The State University of New York, Albany, NY, United States; ^5^Department of Psychology, Pennsylvania State University, University Park, PA, United States; ^6^Department of Psychology, Lawrence University, Appleton, WI, United States

**Keywords:** reproducibility movement, failed replications, researcher gender, career impact, gender stereotypes

## Abstract

The reproducibility movement in psychology has resulted in numerous highly publicized instances of replication failures. The goal of the present work was to investigate people’s reactions to a psychology replication failure vs. success, and to test whether a failure elicits harsher reactions when the researcher is a woman vs. a man. We examined these questions in a pre-registered experiment with a working adult sample, a conceptual replication of that experiment with a student sample, and an analysis of data compiled and posted by a psychology researcher on their public weblog with the stated goal to improve research replicability by rank-ordering psychology researchers by their “estimated false discovery risk.” Participants in the experiments were randomly assigned to read a news article describing a successful vs. failed replication attempt of original work from a male vs. female psychological scientist, and then completed measures of researcher competence, likability, integrity, perceptions of the research, and behavioral intentions for future interactions with the researcher. In both working adult and student samples, analyses consistently yielded large main effects of replication outcome, but no interaction with researcher gender. Likewise, the coding of weblog data posted in July 2021 indicated that 66.3% of the researchers scrutinized were men and 33.8% were women, and their rank-ordering was not correlated with researcher gender. The lack of support for our pre-registered gender-replication hypothesis is, at first glance, encouraging for women researchers’ careers; however, the substantial effect sizes we observed for replication outcome underscore the tremendous negative impact the reproducibility movement can have on psychologists’ careers. We discuss the implications of such negative perceptions and the possible downstream consequences for women in the field that are essential for future study.

## Introduction

Sharing a frustration that I’m working through—one that I think many mobbing/bullying targets have experienced: My organization/field is beginning to acknowledge the existence of a destructive and pervasive cultural problem: harassment, abuse, bullying, and mobbing (Amy Cuddy, PhD, Twitter, April 11, 2021).

And I’m really not saying this to be a jerk. I’ve been on Twitter for 4 years and I’ve seen this over and over and over again. And once again, it’s often targeted toward women scholars (Jide Bamishigbin, PhD, Twitter, November 13, 2021).

Relative to their representation in the natural and physical sciences, women faculty are far better represented in the social and life sciences (e.g., Ginther and Kahn, 2014) and yet continue to face barriers to success. According to data from the National Center for Science and Engineering Statistics Survey of Earned Doctorates, women earned 59.9% of doctoral degrees awarded in psychology and the social sciences in 2020, up from 46.6% in 1990 (National Center for Science and Engineering Statistics Survey of Earned Doctorates (NCSES), 2021). Despite these gains, women academics in the social and life sciences are underrepresented as invited colloquium speakers at prestigious research universities (Nittrouer et al., 2018), and, in social and personality psychology specifically, they are less likely than men to be cited (Brown and Goh, 2016) or have their research included in graduate-level syllabi (Skitka et al., 2021). In a profession where promotion often depends on establishing and sustaining a national research reputation in one’s field of study, such gender disparities should not be taken lightly. Indeed, these factors may help explain why women-identified social scientists remain significantly underrepresented at the level of full professor (e.g., Ginther and Kahn, 2014; National Center for Science and Engineering Statistics Survey of Earned Doctorates (NCSES), 2021), the highest rank within academia. In the present research, we explored another potential mechanism through which women social scientists may be disadvantaged. Specifically, we examined whether women researchers face greater reputational consequences than men when their work fails to replicate and whether they are disproportionately targeted by the reproducibility movement in psychology. Our research questions were inspired by highly publicized cases in psychology in which researchers expressed concerns about failed replications leading to personal mistreatment (as illustrated by the first of our opening quotes) and by anecdotal observations that such mistreatment seems more often directed at women researchers (as the second of our opening quotes suggests). Despite numerous anecdotes, we could find no previously published work to address these important research questions.

## Impact of the Reproducibility Movement on Trust in Psychological Science and Scientists

For over a decade, the field of psychology has experienced a crisis of confidence (see Fanelli, 2018). Some trace the origins of this crisis to a widely cited publication on the surprising prevalence of false positives (i.e., when researchers reject a null hypothesis that is, in fact, true) (Simmons et al., 2011). Subsequently, a revolution of sorts took over the field, with psychological scientists adopting many of the recommendations made by Simmons et al., to achieve greater research transparency. These included tactics such as mechanisms for pre-registration of hypotheses and data analytic procedures, as well as journals’ increasing willingness to publish registered reports for which as-predicted significant results are not required. In addition, the field experienced the launching of large-scale replication projects (e.g., ManyLabs) to assess the reproducibility of widely cited past findings with appropriate statistical power. Unquestionably, open science practices such as pre-registration, increased statistical power in studies, and the publication of replications are best practices in the social sciences. Indeed, replicability is a key ingredient in the scientific process, and increasing transparency of methods and analyses is a welcome advancement in scientific norms (e.g., Asendorpf et al., 2013).

Although replication is a critical part of the scientific method, some researchers have argued that the “reproducibility crisis narrative” is an unnecessarily dramatic description of the problem (Fanelli, 2018). There are myriad reasons why scientific findings may fail to replicate (Asendorpf et al., 2013; Maxwell et al., 2015; Schmidt and Oh, 2016), and many of these have little to do with the scientific competence or ethical research practices of individual researchers. Rates of engagement in outright scientific misconduct and (perhaps less egregious but still problematic) questionable research practices (QRPs) are likely relatively low (Fanelli, 2009). Rather, replicability of findings in psychology depends on the contextual sensitivity of the research topic, above and beyond statistical power and effect size (Van Bavel et al., 2016), and replication efforts led by less experienced teams of researchers are more likely to fail than those led by teams with greater research expertise (Bench et al., 2017). Furthermore, one systematic analysis of social science experiments published in *Nature* and *Science* over a 5 year period revealed that social scientists’ *beliefs* about the replicability of a study’s findings predicted their actual replicability (Camerer et al., 2018). Specifically, researchers were provided copies of 21 replication reports and citations for the originally published studies prior to the conduct of the replications, and they were asked to predict the likelihood that each of the studies selected for the large-scale replication project would successfully replicate. Results revealed that researchers’ aggregated predictions about a given study’s replicability strongly predicted the outcome of the associated replication, *r* = 0.76. On the one hand, this finding may suggest that researchers are able to identify conditions under which findings may not successfully replicate with surprising accuracy; but, on the other hand, this finding also suggests that replication outcomes are not solely due to chance. It could be that, based on their beliefs about a given study’s replicability, researchers sometimes “cherry-pick” which studies to target for replication, which could artificially inflate the rates of failed replications. Of note, systematic investigations of replication studies suggest that the majority provide weak or inconclusive evidence for the reproducibility of the original findings (Etz and Vandekerckhove, 2016). In short, a single failed replication is inconclusive, and yet, the number of Web of Science papers perpetuating the “crisis” narrative exponentially increased in the late 2010s (Fanelli, 2018).

Several studies suggest that the increased salience of the reproducibility movement may pose some serious consequences for the reputation of the field of psychology. In one study, reading about replication failures in psychology (relative to a control condition in which participants read about psychological research in general), decreased trust in past research in psychology (Anvari and Lakens, 2018). In that same study, exposure to information about replication failures was no different from exposure to information about QRPs in psychology, and reading about reforms to address psychology’s reproducibility problem actually served to undermine participants’ trust in future psychological research. Wingen et al. (2020) conceptually replicated those findings, not only demonstrating that learning about low replicability rates in psychology decreases public trust in the field, but also demonstrating that commonly used strategies to repair trust (e.g., increased transparency in research methods reporting) did not significantly restore it. Although there are various reasons for failed replications, these studies suggest that many people do not distinguish among them. Indeed, relative to their baseline attitudes, even a one-hour lecture on the replication crisis that explicated the many reasons for replication failure, from low statistical power to fraudulent research practices, decreased undergraduate students’ trust in psychology research results as measured post-lecture (Chopik et al., 2018).

Although failures to replicate do not necessarily imply either scientific misconduct or incompetence, studies have revealed that researchers fear personal reputational consequences of failed replications. For example, in one study, researchers were asked to imagine one of their own findings versus someone else’s findings failing to replicate, and what the reputational costs would be. Consistently, these researchers believed that their own reputation (both scientific and social) would suffer more, and that their work (both their original finding and other work from their lab) would be perceived more negatively (Fetterman and Sassenberg, 2015). Although the authors concluded from their findings that researchers overestimate the reputational costs of failed replications, their data clearly demonstrate that researchers are concerned about how they will be perceived should their studies fail to replicate.

Some studies suggest that researchers’ concerns about the reputational impact of failed replication attempts may be well-founded. Ebersole et al. (2016) asked United States survey respondents first to imagine and evaluate a researcher who found and published an interesting result. Respondents were then asked to evaluate the same researcher when another researcher successfully replicated the target researcher’s interesting results, and then later to evaluate that same researcher when another researcher failed to replicate the interesting results. Relative to the control condition with no information about the results’ replicability, perceptions of the researcher’s ability and ethics, as well as perceived truthfulness of the results, increased when another researcher replicated the original findings, but significantly decreased when the findings failed to replicate. Similarly, in a primarily German sample, Hendriks et al. (2020) manipulated whether a study replication attempt was successful or unsuccessful, and found that failure to replicate decreased ratings of study credibility and researcher trustworthiness.

Collectively, these studies demonstrate that failed replications can have negative consequences for perceptions of both the field and individual researchers. To date, key outcomes of extant experiments have been limited to perceptions of researcher ability, ethics, credibility and trustworthiness, as well as truthfulness or trustworthiness of study results; these are important outcomes given that people do not always appreciate the differences between QRPs and other factors that affect replicability of a single study (e.g., Chopik et al., 2018). In the current research, we conceptually replicated these past experiments and also expanded them to include broader perceptions of the researcher, such as their likability and intentions to interact with the researcher or with their work in the future, as well as perceptions of the importance of their research. These outcomes speak to other reputational costs that may affect researchers’ careers, such as whether they get invited as a consultant in applied settings and whether they are able to attract students to assist in their labs. Consistent with extant research, we expected that, relative to a successful replication attempt, a researcher whose original findings failed to replicate would be perceived as less competent, likable, lower in integrity, and less likely to elicit a desire for future interactions, and that their research would be considered less important and fundable.

## The Possible Backlash Against Women Social Scientists

Simmons et al. (2011) indicated, “Our goal as scientists is not to publish as many articles as we can, but to discover and disseminate truth” (p. 1365). But in their very next sentence, the authors go on to admit that even they themselves “often lose sight of this goal, yielding to the pressure to do whatever is justifiable to compile a set of studies that we can publish.” In fact, in an investigation of publication trends in social psychology, Sassenberg and Ditrich (2019) found that the number of studies per article in the field’s top journals significantly increased following the Simmons et al. call to action, as did the average sample size per study. However, these practices came at the expense of laboratory investigations and behavioral measures that are more time-intensive and effortful to conduct, but that were once considered a hallmark of social psychological research (e.g., Baumeister et al., 2007). For better or for worse, researchers are clearly changing their methods in response to increased pressure to conduct and replicate highly powered studies.

Such pressures to “do whatever it takes” to succeed are consistent with a masculinity contest culture, a culture in which ambition, independence, and assertiveness (characteristics of agency and dominance) are valued, and sensitivity and vulnerability (characteristics of communality) are disparaged (Berdahl et al., 2018; Glick et al., 2018). If social science research has become a masculinity contest culture, as some have suggested about academia more broadly (Kaeppel et al., 2020), then researchers of all genders are at greater risk of burnout, job dissatisfaction, and experiences with harassment (Glick et al., 2018). Furthermore, if behaviors associated with agentic dominance are rewarded more than those associated with communality in social science research, then women researchers who assert themselves in efforts to succeed are at increased risk of backlash (Rudman et al., 2012). Indeed, meta-analytic evidence demonstrates that women are penalized more than men for dominance displays, both in terms of their likability and downstream career consequences such as hireability (Williams and Tiedens, 2016).

Psychology provides a unique context for examining potential backlash against women researchers, given that, in this field, women outnumber men (albeit at lower ranks; Ginther and Kahn, 2014; National Center for Science and Engineering Statistics Survey of Earned Doctorates (NCSES), 2021), and given that the field is stereotypically associated with feminine traits to a greater degree than with masculine traits (Boysen et al., 2021). Thus, one might expect greater equity in psychology and other social sciences than in the physical and natural sciences. In fact, one study found a 2:1 preference for women candidates over equally qualified men with regard to hiring at the assistant professor level in psychology (as well as biology, engineering, and economics; Williams and Ceci, 2015). Furthermore, some evidence suggests that previously observed barriers for women’s advancement are beginning to break down. For example, women applying for a position who were once described more by communal traits in their letters of reference—which predicted lower ratings of hireability (Madera et al., 2009)—are now described similarly as their male counterparts, and in some cases are described more positively than men (Bernstein et al., 2022). This is good news for women applying to academic positions, as their odds of being hired may be improving (see also Ceci, 2018).

Despite some evidence of increasing gender equity, other findings suggest that women social scientists do not always experience more equitable or favorable treatment once in the field itself. For example, women hold significantly fewer positions of power within psychology (Gruber et al., 2021), which may limit their ability to advocate for certain gender-equity practices that women administrators value more than men, such as accommodations for mothers in federal grant funding (Williams et al., 2017). In addition, in social psychology (a field which, according to membership in its largest professional organization, is 51% female), women comprised only 34% of first authors in a random sample of issues of the field’s flagship journal in a 10-year time period, were significantly less likely to be cited than men, and received only 25% of the society’s top professional awards (Brown and Goh, 2016). Another examination of social psychology’s largest conference revealed that women were significantly underrepresented as speakers, and this was especially true for women lower in academic rank (Johnson et al., 2017). Similarly, women in psychology and other fields with relative gender parity were significantly less likely than men to give invited colloquia at top research universities (Nittrouer et al., 2018). Such findings likely have downstream implications for women’s careers in academic psychology, given the importance of lead authorship in top journals and conference symposia, of receiving national awards, and of giving invited talks at prestigious institutions in promotion and tenure decisions.

Although many of the aforementioned findings are descriptive in nature, they are corroborated by past and recent experimental evidence (e.g., Steinpreis et al., 1999; Heilman and Haynes, 2005; Moss-Racusin et al., 2012; Proudfoot et al., 2015; Bian et al., 2018; Régner et al., 2019; Witteman et al., 2019; Begeny et al., 2020). For example, when abstract submissions to an international social science conference were manipulated with male-typical versus female-typical names, not only were supposedly male-authored abstracts viewed as higher quality, but when the male-authored abstract featured stereotypically more masculine research topics, the research was especially likely to yield high ratings of quality (Knobloch-Westerwick et al., 2013). Similarly, psychological research journals that are gender-related (vs. other specialty journals) are viewed as less meritorious even when they have the same technical impact factor (Brown et al., 2022). Collectively, these findings suggest the greater perceived value of masculinized knowledge, even in the stereotypically feminine (Boysen et al., 2021) field of psychology (see also Niemann et al., 2020). In a profession arguably governed by masculine defaults (Cheryan and Markus, 2020), we therefore expected that any replication failure would be viewed negatively, and that, in particular, social science women whose research findings fail to replicate would be evaluated more critically and negatively than men.

## Overview of the Current Research

Although anecdotal data (such as the Tweets quoted in the introduction to this paper) point toward the possibility that women are targeted more harshly and/or more often than men by the reproducibility movement, we could find no prior efforts to investigate this systematically. The goal of the present work was to address this important limitation and to expand upon the nascent knowledge base regarding perceptions of failed replications in psychology. First, we wished to examine a broad range of reactions to a researcher’s (ostensible) replication failure versus success, replicating and extending past experiments documenting some narrower reputational costs of failed replications (Ebersole et al., 2016; Hendriks et al., 2020). We explored both attitudes and behavioral intentions toward interacting with the researcher, as well as perceptions of their research in general, and we predicted an overall replication-failure bias (such that researchers and their work broadly would be perceived substantially more negatively in the replication failure vs. success condition). Second, we investigated the gender-replication hypothesis; we expected that, relative to men, women researchers would not only be evaluated more negatively for failed replications, but also targeted more often in reproducibility efforts.

We examined these research questions in a pre-registered experiment conducted with a working adult sample, a replication of that experiment conducted with a student sample, and in an analysis of archival data from one public website. Participants in the experiments were randomly assigned to read a (fictional) news article describing a successful versus failed replication attempt of social science research, in which the author’s name and pronouns were manipulated to portray a woman or man psychological scientist. We also culled researcher gender data from a website that portrays itself as dedicated to improving research replicability to examine the proportion of men and women “targets” of replication tests.

## Study 1

We were first interested in public reactions to a failed social science replication, and whether those reactions might vary as a function of researcher gender. Public perceptions were of interest for a number of reasons. First, psychology’s reproducibility “crisis” was highly publicized, with news stories appearing in popular media outlets, countless social media posts casting doubt on classic and/or intriguing or “sexy” findings (e.g., such as Dr. Amy Cuddy’s power pose findings referenced in the opening quote), and “watchdog” websites being launched to monitor scientific replications and retractions. Second, the reproducibility movement appeared to converge with an increasing public distrust of scientific experts in the United States, particularly among political conservatives (Pew Research Center, 2019), thereby potentially constituting a source of divisiveness among voters. Finally, and most germane to our purposes in the present research, public perceptions of research, perhaps especially when politicized, can have serious career consequences for researchers whose work has caught the public eye (for an example, see news stories regarding Dr. Nikole Hannah-Jones’ tenure rejection; e.g., Folkenflik, 2021).

### Method

#### Participants

Adult participants at least 18 years of age were recruited from Amazon’s Mechanical Turk through CloudResearch’s (formerly TurkPrime) MTurk Toolkit (Litman et al., 2017). Participants were compensated with $1 USD. Four hundred and twelve individuals opened the study link, but 360 people actually participated in the study. This number was larger than our pre-registered target sample size of 320, which was based on recommendations of at least 40 participants per cell. G*Power software (Faul et al., 2009) furthermore indicated that a sample of 199 participants would provide 80% power to test our hypothesized interaction, assuming a small effect size (*d* = 0.20) and an alpha of 0.05. After removing 15 participants (4.17% of the sample) who failed at least one of two attention check items, 55 (15.28%) who failed the scientist gender manipulation check (24 in the male scientist condition and 31 in the female scientist condition), and 24 participants (6.67%) who failed the replication outcome manipulation check (11 in the successful condition, 13 in the failed condition), the working sample included 266 (mostly White, 77.1%) participants (149 men, 117 women) with an average age of 35.03 years (*SD* = 11.06 years). Participants represented all regions of the United States, and the vast majority (96.2%) were originally from the United States, with English as their first language (95.9%). Most (83.8%) were not students, but rather were employed in or retired from various occupations (e.g., retail, business, computing). Participants overall were very unfamiliar with the Strack facial feedback research prior to participation (*M* = 2.21, *SD* = 1.57), which was significantly below the scale midpoint of 4, *t*(265) = 18.59, *p* < 0.001, *d* = 1.14.

#### Design and Procedure

This study was pre-registered at https://osf.io/vy246/?view_only=95879962e6fb469fb226157edaecd861. The experiment employed a 2 (replication outcome: successful vs. failed replication) × 2 (scientist gender: male vs. female) between-subjects design. After consenting to participate, participants were told that researchers were interested in the public’s perceptions of scholars whose research is part of the “reproducibility project.” Introductory information defined and explained the purpose of replication and its importance to science. In addition, the information explained that sometimes research replicates and sometimes it does not. Participants then were required to spend at least 2 min viewing an ostensibly real single-page science news article describing the outcome of a large-scale attempt to replicate the original experimental investigation of the facial feedback hypothesis by Strack et al. (1988), in which holding a pen between their teeth (i.e., forcing a smile) elevated participants’ mood, whereas holding a pen between their lips (i.e., forcing a frown) worsened participants’ mood. This article contained our experimental manipulations. Participants were randomly assigned to one of four resulting conditions in which the experiment of Dr. Brian Strack versus Dr. Karen Strack either replicated or failed to replicate. They then completed (in a random order) measures of the researcher’s competence, likability, integrity, perceptions of the research, and desired future interactions with the researcher. For each item that referred to the researcher, the researcher’s first name (i.e., Brian or Karen) was piped in to ensure the salience of the researcher gender manipulation.^1^ In addition, instructions for each of the key dependent measures underscored that we were interested in participants’ opinions and that there were no right or wrong answers. Two attention check items were embedded in the dependent measures (i.e., “If you are reading this, click the number 4″; “The answer to this question is 2. Please click 2.”). Following their completion of the dependent measures (described in greater detail below), participants responded to two manipulation check items to ensure that they could correctly identify the gender of the researcher from the news story (i.e., “What was the gender of the researcher you read about?”) and the outcome of the replication attempt (i.e., “What was the outcome of the replication attempt that you read about?”). Lastly, participants completed demographic items, including gender, race and ethnicity, age, United States region of residence, country of origin, whether English is their first language, socioeconomic status, occupation, and student status. Participants also completed an item to assess their degree of familiarity with psychological research on the effects of smiling on mood prior to taking part in the study (1 = not at all familiar to 7 = extremely familiar). The last screen of the survey debriefed participants with regard to the purpose of the study and the fact that the news article was created for the purposes of the experiment.

#### Measures

##### Researcher Competence

Participants completed 12 items assessing perceived researcher competence. Seven of the items, adapted from Smith et al. (2007), were statements (e.g., I would describe Dr. Strack as a highly skilled researcher) to which participants indicated their agreement on seven-point Likert-type scales (1 = strongly disagree to 7 = strongly agree). Four items, adapted from Moss-Racusin and Miller (2016) asked participants to indicate the likelihood (1 = not at all to 7 = very much) that Dr. Strack had certain qualities (e.g., the necessary skills to perform well as a researcher). One item asked participants to indicate the likelihood (on a scale from 0% no chance to 100% definitely) that Dr. Strack would receive a prestigious award for their research in the next 5 years. After reverse-scoring relevant items, we standardized each, given they were on different scales, and examined their reliability. The 12 standardized items were highly reliable (α = 0.92) and were averaged to form a scale, with higher scores indicating greater perceived competence.

##### Researcher Likability

Using a seven-point scale (1 = not at all to 7 = very much), participants responded to six items adapted from Smith et al. (2007) and Moss-Racusin and Johnson (2016) regarding how much they thought they would like the researcher. For example, “I think I would like Dr. Strack as a person.” The items demonstrated high internal consistency (α = 0.92) and were averaged to form an index, with higher scores indicating greater likability.

##### Researcher Integrity

Participants indicated on seven-point scales (1 = not at all to 7 = very much) the extent to which they perceived the researcher as having integrity. There were nine total items (e.g., “To what extent do you think Dr. Strack is trustworthy?”). Three of these items were adapted from Biernat et al. (1996), one item was adapted from Smith et al. (2007), and the remaining five items were created for this study. After reverse-scoring relevant items, reliability analysis indicated strong internal consistency (α = 0.94). We created an index of researcher integrity by averaging items such that higher scores indicate greater perceived integrity.

##### Perceptions of the Research

Participants responded to seven items concerning their perceptions of the research. Two items were adapted from Handley et al. (2015) and concerned funding, including one open-ended question regarding the budget they would suggest Dr. Strack should receive for more research in this area by the National Foundation. Participants were told that such grants typically range from $100,000 to $900,000 with an average of $500,000. Participants responded to the remaining items (e.g., How important is more research on this topic) on seven-point scales (1 = not at all to 7 = very much). After reverse-scoring relevant items, items were standardized, as they were on different scales. The standardized items demonstrated good reliability (α = 0.91) and were averaged to form an index with higher scores indicating more favorable perceptions of the research.

##### Future Interactions

Participants responded to five items (e.g., How likely are you to attend a public lecture by Dr. Strack) regarding their desired future interactions with the researcher. The items were developed for this study based on ways that the public might likely engage with academic researchers (e.g., attending lectures, searching for additional articles by the researcher). Participants responded using seven-point scales (1 = extremely unlikely to 7 = extremely likely). The items were highly reliable (α = 0.91) and were averaged to form an index such that higher scores represent greater likelihood of future interactions.^2^

### Results and Discussion

We first examined the descriptive statistics and correlations among the variables of interest. As shown in Table 1, outcomes were positively correlated, as expected.

**Table 1:** Descriptives and correlations among all outcomes in Study 1.

	Mean (SD)	1	2	3	4	5	6	7	8	9
1. Standardized competence	0.00 (0.74)	(0.92)								
2. Likelihood prestigious award	35.93 (27.74)	0.63^*^	−							
3. Competence (sans Award item)	4.92 (1.06)	0.99^*^	0.56^*^	(0.92)						
4. Likability	4.38 (1.29)	0.64^*^	0.43^*^	0.64^*^	(0.92)		.			
5. Integrity	4.57 (1.29)	0.76^*^	0.40^*^	0.76^*^	0.72^*^	(0.94)				
6. Standardized research perceptions	0.00 (0.80)	0.79^*^	0.56^*^	0.78^*^	0.68^*^	0.73^*^	(0.88)			
7. NSF	239859.78 (204,587.04)	0.59^*^	0.44^*^	0.58^*^	0.45^*^	0.53^*^	0.72^*^	−		
8. Research perceptions (sans NSF item)	4.45 (1.20)	0.81^*^	0.52^*^	0.81^*^	0.64^*^	0.79^*^	0.93^*^	0.60^*^	(0.87)	
9. Future interactions	3.48 (1.67)	0.60^*^	0.52^*^	0.58^*^	0.65^*^	0.61^*^	0.75^*^	0.52^*^	0.67^*^	(0.91)

* Correlation is significant at the 0.01 level (two-tailed).

Cronbach’s alpha is reported on the diagonal where relevant. Competence and Research Perceptions were standardized to accommodate different scales of measurement. Likelihood of Prestigious Award was a single item (0–100) that was part of the Standardized Competence scale, and Competence sans this item is reported for interpretation of mean scores. NSF was a single item numeric response that could range from 0 to 900,000 and was part of the Standardized Research Perceptions scale. All other measures were on seven-point scales.

Next, we conducted univariate analyses of variance (ANOVAs) on each dependent measure, including participant gender as a variable. Across all of these analyses, participant gender yielded only two significant findings, and neither qualified any of the findings reported below; thus, we dropped participant gender from analyses and report findings from two-way between-subjects ANOVAs including scientist gender and replication outcome as predictors. Furthermore, controlling for participants’ self-reported prior familiarity with Strack’s facial feedback research did not change any of the reported results.

Results revealed significant main effects of replication outcome on each of the dependent variables. As shown in Table 2, participants perceived the researcher as significantly less competent [*F*(1, 262) = 63.53, *p* < 0.001, *d* = 0.98], less likable [*F*(1, 262) = 21.27, *p* < 0.001, *d* = 0.57], and as having less integrity [*F*(1, 262) = 55.35, *p* < 0.001, *d* = 0.91] when their work failed to replicate than when it successfully replicated. Additionally, participants perceived the research less favorably (e.g., as less important and deserving of funding) [*F*(1, 262) = 62.12, *p* < 0.001, *d* = 0.97], and intended to interact less with the researcher [*F*(1, 261) = 53.17, *p* < 0.001, *d* = 0.90] when their results failed to replicate than when the replication attempt was successful. Across study outcomes, there were no significant main effects of nor interactions with scientist gender (*p*s > 0.05), in contrast to our gender-replication hypothesis.

**Table 2:** Study 1 dependent variable means and standard deviations by experimental conditions and participant gender.

	Successful replication				Failed replication			
	Male scientist		Female scientist		Male scientist		Female scientist	
	Men	Women	Men	Women	Men	Women	Men	Women
Competence	0.30 (0.50)	0.38 (0.52)	0.17 (0.75)	−0.27 (0.70)	−0.30 (0.81)	−0.26 (0.62)	−0.46 (0.72)	−0.27 (0.70)
Likability	4.72 (1.35)	4.34 (1.43)	4.55 (1.25)	5.23 (1.14)	4.17 (1.12)	3.95 (1.32)	3.79 (1.13)	4.17 (1.11)
Integrity	4.98 (1.28)	4.80 (1.20)	5.17 (1.12)	5.43 (0.95)	4.02 (1.22)	4.22 (1.14)	3.77 (1.04)	4.17 (1.34)
Research Perceptions	0.35 (0.77)	0.21 (0.82)	0.22 (0.67)	0.59 (0.66)	−0.39 (0.61)	−0.18 (0.75)	−0.54 (0.71)	−0.27 (0.75)
Future Interactions	4.26 (1.71)	3.84 (1.71)	4.02 (1.39)	4.41 (1.63)	2.88 (1.39)	2.89 (1.48)	2.63 (1.32)	2.75 (1.64)

Competence and Research Perceptions were standardized to accommodate different scales of measurement. All other measures were on seven-point scales.

Study 1 conceptually replicated past research and provided further evidence that replication failures lead to more negative perceptions of researchers and their research. Expanding upon earlier findings (Ebersole et al., 2016; Hendriks et al., 2020), we demonstrated in a sample of adults not only did the public have more negative perceptions of a researcher’s competence and scientific integrity and of their research when their findings failed to replicate than when they replicated successfully, but they also liked the researcher less and reported weaker behavioral intentions to interact with them in the future. Of importance, the observed effect sizes were very robust, suggesting that the consequences of a failed replication are quite serious and arguably greater than is justified, given the nature of what a failed replication can(not) tell us (Maxwell et al., 2015; Schmidt and Oh, 2016).

Contrary to our gender-replication hypothesis, findings did not suggest that women were evaluated more harshly than men when their findings failed to replicate. We considered, however, whether that was a function of the sample of laypeople, who are perhaps less invested than some other populations in evaluating academic psychologists, with whom they may have limited personal interactions. Might people who engage more regularly with psychology faculty, such as college students, respond differently? We conducted the same experiment with a sample of undergraduate students to explore that possibility.

## Study 2

Some research provides reason to believe that college undergraduates would be more critical of women faculty whose work fails to replicate than they would be of a male faculty member with a failed replication. For example, in an experiment of teaching evaluations, students in a social science course were randomly assigned to online discussion groups in which a male versus a female assistant presented themselves with their own versus the other assistant’s identity (i.e., as male vs. female, regardless of their own gender). At the end of the term, students evaluated their assistant instructor more harshly when they perceived her to be female (MacNell et al., 2015). In other research, women-identifying professors not only reported experiencing more requests for special favors from students than their male colleagues, but experimental evidence also demonstrated that students were more likely to expect a female vs. a male professor to grant favors, especially when those students were high in academic entitlement (El-Alayli et al., 2018). These studies collectively suggest that women faculty may walk a tighter rope with students than male faculty walk (see Williams and Dempsey, 2014). Thus, we again tested our gender-replication hypothesis, but with a student sample.

### Method

#### Participants

The design of the experiment was identical to that of Study 1, for which G*Power software (Faul et al., 2009) recommended a sample of 199 participants would provide 80% power to test our hypothesized interaction, assuming a small effect size (*d* = 0.20) and an alpha of 0.05. Given we did not obtain the predicted interaction in Study 1, we intentionally increased our sample in Study 2. Three hundred fifty students enrolled in Introduction to Psychology at a large Midwestern university completed the study in exchange for research credit. After removing 17 participants (4.86% of the sample) who failed the scientist gender manipulation check (13 in the male scientist condition and 4 in the female scientist condition), and 43 participants (12.29% of the sample) who failed the replication outcome manipulation check (22 in the successful condition, 21 in the failed condition), 19 people (5.43%) who were missing manipulation check data, and another 19 (5.43%) with missing or failed attention check data, the working sample included 252 (mostly White, 74.2%) participants (139 men, 111 women, 2 other) with an average age of 20.31 years (SD = 3.93 years). Most participants (89.3%) were originally from the United States, with English as their first language (87.3%) and middle-class self-reported SES (*M* = 3.44, *SD* = 1.06 on a 5-point scale where 1 = I cannot make ends meet to 5 = I do not have to worry about money). Participants overall were not familiar with the Strack facial feedback research prior to participation (*M* = 3.35, *SD* = 1.94), which was significantly below the scale midpoint of 4, *t*(251) = 5.29, *p* < 0.001, *d* = 0.33.

#### Design and Procedure

The experimental design and procedure were identical to Study 1.

### Measures

Measures were identical to those used in Study 1, with the exception of the future interaction items, which were adapted to fit the ways in which college students, instead of the general public, might interact with researchers (e.g., take a class with Dr. Strack, apply to work in Dr. Strack’s lab as a research assistant). In this study, seven items were used to assess likelihood of future interactions, and as in Study 1, these items were highly reliable (α = 0.89) and were therefore averaged to form an index where higher scores indicate greater likelihood of future interactions with the scientist. Researcher competence (α = 0.89), likability (α = 0.88), integrity (α = 0.90), and perceptions of the research (α = 0.85) were identical to the measures used in Study 1 and had similarly good psychometric properties.

### Results and Discussion

Similar to Study 1, dependent variables were positively correlated, as shown in Table 3. In addition, analyses revealed inconsistent main effects of participant gender, but in no case did participant gender interact with the key manipulation of replication outcome. Thus, we dropped it from further analysis and report main effects of and interactions between scientist gender and replication outcome for each of the dependent variables. Additionally, controlling for participants’ familiarity with prior research on the facial feedback hypothesis did not change our findings.

**Table 3:** Descriptives and correlations among all outcomes in Study 2.

	Mean (SD)	1	2	3	4	5	6	7	8	9
1. Standardized competence	0.03 (0.66)	(0.89)								
2. Likelihood prestigious award	38.16 (25.34)	0.59^*^	−							
3. Competence (sans Award item)	4.76 (0.79)	0.99^*^	0.50^*^	(0.88)						
4. Likability	3.92 (1.06)	0.51^*^	0.33^*^	0.51^*^	(0.88)		.			
5. Integrity	4.43 (1.09)	0.66^*^	0.38^*^	0.66^*^	0.59^*^	(0.90)				
6. Standardized research perceptions	0.03 (0.72)	0.65^*^	0.44^*^	0.64^*^	0.58^*^	0.57^*^	(0.85)			
7. NSF	265858.96 (176,845.38)	0.42^*^	0.38^*^	0.41^*^	0.31^*^	0.33^*^	0.64^*^	−		
8. Research perceptions (sans NSF item)	4.17 (1.11)	0.64^*^	0.41^*^	0.63^*^	0.58^*^	0.56^*^	0.99^*^	0.50^*^	(0.84)	
9. Future interactions	3.42 (1.37)	0.52^*^	0.45^*^	0.50^*^	0.52^*^	0.54^*^	0.59^*^	0.32^*^	0.59^*^	(0.89)

* Correlation is significant at the 0.01 level (two-tailed).

Cronbach’s alpha is reported on the diagonal where relevant. Competence and Research Perceptions were standardized to accommodate different scales of measurement. Likelihood of Prestigious Award was a single item (0–100) that was part of the Standardized Competence scale, and Competence sans this item is reported for interpretation of mean scores. NSF was a single item numeric response that could range from 0 to 900,000 and was part of the Standardized Research Perceptions scale. All other measures were on seven-point scales.

Replicating findings from Study 1, results revealed significant main effects of replication outcome on each of the dependent variables, but no main effect of or interaction with scientist gender (*p*s > 0.23). As shown in Table 4, participants perceived the researcher as significantly less competent [*F*(1, 248) = 27.59, *p* < 0.001, *d* = 0.66], less likable [*F*(1, 248) = 21.34, *p* < 0.001, *d* = 0.58], and as having less integrity [*F*(1, 248) = 51.87, *p* < 0.001, *d* = 0.91] when their work failed to replicate than when it successfully replicated. Participants also perceived the research less favorably [*F*(1, 248) = 27.16, *p* < 0.001, *d* = 0.66], and indicated poorer likelihood of future interactions with the researcher [*F*(1, 248) = 34.56, *p* < 0.001, *d* = 0.74] when the replication attempt was unsuccessful compared with when it was successful.

**Table 4:** Study 2 dependent variable means and standard deviations by experimental conditions and participant gender.

	Successful replication				Failed replication			
	Male scientist		Female scientist		Male scientist		Female scientist	
	Men	Women	Men	Women	Men	Women	Men	Women
Competence	0.14 (0.49)	0.29 (0.57)	0.09 (0.54)	0.41 (0.61)	−0.29 (0.53)	−0.05 (0.71)	−0.26 (0.83)	−0.08 (0.70)
Likability	4.30 (1.08)	4.26 (1.01)	3.90 (1.08)	4.36 (0.97)	3.58 (0.88)	3.63 (0.93)	3.53 (1.02)	3.71 (1.22)
Integrity	4.66 (0.95)	4.94 (0.99)	4.78 (1.17)	5.17 (0.94)	3.83 (0.79)	4.13 (0.87)	3.75 (1.06)	4.24 (1.09)
Research Perceptions	0.23 (0.72)	0.21 (0.66)	0.17 (0.57)	0.47 (0.60)	−0.21 (0.68)	0.03 (0.67)	−0.38 (0.66)	−0.16 (0.79)
Future Interactions	3.47 (1.47)	4.18 (1.17)	3.77 (0.97)	4.38 (1.35)	3.01 (1.16)	2.83 (1.31)	2.67 (1.31)	3.14 (1.23)

Competence and Research Perceptions were standardized to accommodate different scales of measurement. All other measures were on seven-point scales.

Demonstrating the generalizability of Study 1 findings across different populations, Study 2 further reinforced the extent to which failed replications in psychology affect confidence in both researchers and their research. These findings are potentially costly for academic researchers’ career advancement, given the important role that students play in faculty promotion and tenure. For example, many social science faculty depend on undergraduate students as research assistants, and oftentimes can use students’ accomplishments (e.g., research products, admissions to graduate programs) as evidence of their impact as a mentor. To the extent that failed replications raise doubts about researchers’ competence and integrity, and decrease students’ likelihood of taking classes from or seeking opportunities to work with them, faculty performance reviews will likely suffer.

As in Study 1, we did not find support for our gender-replication hypothesis in this study. Students were equally critical of a male versus a female researcher whose work failed to replicate, and equally favorable of those whose work successfully replicated. This was surprising, in light of previous studies in which students more negatively evaluated or expected more from female faculty versus male faculty (e.g., MacNell et al., 2015; El-Alayli et al., 2018). On the other hand, information about a failed replication may not make salient the fact that faculty not only conduct research, but also, as teachers and mentors, are frequently in positions in which they are critical of students, which is a key driver of students’ denigration of women faculty (e.g., Sinclair and Kunda, 2000).

In Study 3, we tested our gender-replication hypothesis with a different method: archival analysis of data from a public replication-monitoring website. Although our data thus far suggest that women researchers are not evaluated more harshly than men researchers when their work fails to replicate, it could be that women are targeted more often in replication efforts.

## Study 3

The effort to document the replicability of studies in psychology has led to the establishment of a variety of repositories in which scientists and consumers alike can read about replication results. One example of a popular public website is the Replicability-Index, or R-Index, blog,^3^ created in 2014. Inspired by a controversial publication by social psychologist Bem (2011), the site indicates that its goals are to increase reproducibility of findings in social and personality psychology and to inform consumers of psychological research to problematic publications. Although transparent information-sharing is a welcome change in psychological research practices since the site was developed, some aspects of this particular website are potentially problematic. The site maintains a list of 400 social and personality psychologists who have published in 40 journals identified for analysis without clear selection criteria. Moreover, the complete works of each psychologist appearing on the list were not investigated; again, findings are included based on unspecified criteria. The psychologists are rank-ordered by the extent to which their observed discovery rates match their estimated discovery rates using a z-curve statistical package made available on the site. To be fair, the site points out that results are preliminary and should be interpreted with caution, given they are limited by the specific journals searched and the way results are reported, among “many other factors.” Although helping the public think more critically about psychological (and other) research and increasing accountability among scientists are laudable goals that can serve to improve science, we suggest that targeting individual social scientists in this way (i.e., through a public rank-ordered list with unclear criteria) is counterproductive and invites the kinds of personal attacks described by Dr. Cuddy in our opening quote. In fact, the paragraph preceding the rank-ordered list of psychologists selected for scrutiny on the site states:

“Here I am starting a project to list examples of bad scientific behaviors. Hopefully, more scientists will take the time to hold their colleagues accountable for ethical behavior in citations. They can even do so by posting anonymously on the PubPeer comment site.”

Though this is only one exemplar case of an internet forum on this topic, because the criteria for selecting scientists for this published list were not clearly defined, and people can anonymously nominate scientists for investigation, we suggest that these rather opaque conditions are ripe for gender bias and selected it as a strong case to test our hypothesis. Past research in employment selection demonstrates that gender biases are more likely to manifest themselves when criteria are ambiguous (e.g., Heilman, 2012), and anonymity has been shown to be a key motivator of gender-based harassment (Wesselmann and Kelly, 2010). We therefore examined whether women psychological scientists were overrepresented relative to men on the R-Index site, and whether women were more likely than men to have poor rankings.

### Method

We examined the list of 400 psychologists as it appeared on the R-index website^4^ on 26 July 2021. Two independent coders naïve to the study hypothesis recorded the researchers’ names and replicability rank order as listed on the website, and then they coded each researcher for their gender and indicated the quartile within which they were ranked. Although perceived gender is an imperfect measure of gender, the coders corroborated their ratings with researchers’ websites to the extent that such information was available (e.g., whether the researcher used pronouns on their site), yielding 100% agreement. Chi-square calculations were computed using Preacher (2001) goodness of fit calculation software.

### Results and Discussion

Of the 400 researchers listed on the site, 265 (66.3%) were coded as male and 135 (33.8%) were coded as female. A non-parametric bivariate correlation analysis was conducted using rank order and researcher gender, revealing no significant relationship, Spearman’s rho = −0.02, *p* > 0.66. Thus, on the list as it appeared when data were collected, gender was not associated with rank-ordering by research replicability.

Interpreting these data is extremely challenging, because the criteria for selection are nebulous, and it is difficult to identify the most appropriate comparison for reference. If we assume that the population of social and personality psychologists is half male and half female, roughly reflecting the United States population (United States Census Bureau, 2021), then men are overrepresented in the R-Index list, χ^2^ (1) = 10.56, *p* < 0.01. If we use the most recent membership statistics reported by the Society for Personality and Social Psychology (SPSP, 2019), in which cisgender women make up 54% and cisgender men 41% of the organization, then men appear to be overrepresented to an even greater degree, χ^2^ (1) = 137.11, *p* < 0.001. This might be an appropriate metric if all social and personality psychologists were ranked on the site, but they are not. It is unclear what the “expected percent” of women on a list like this should ultimately be, because so little is known about the criteria that predict having one’s work selected for replication attempts. For example, perhaps it would be more accurate to compare the observed percent of women on the R-Index list to the percent of those who first author “classic” or canonical work in social psychology (assuming that this is the work most likely to be selected for replication attempts, although this may not be the case; see Lindsay, 2015). Although it is difficult to calculate this expected percentage (i.e., operationalizing “canonical” work could be accomplished in many different ways), if we use findings regarding first authorship in social and personality psychology’s top journals, wherein Brown and Goh (2016) reported that 34% were women, then our findings match almost perfectly, χ^2^(1) = 0.003, *p* > 0.95.

Regardless of which existing point of comparison is used, these data suggest that women researchers in social and personality psychology were not overtly targeted by this site more often or ranked lower than their male peers. Thus, we did not find support for our gender-replication hypothesis. That said, given our experimental findings about public and student reactions to researchers whose findings have failed to replicate, coupled with the lack of clarity for how researchers are selected for this site, we maintain that this public list is likely to have reputational costs for the social scientists who appear on it (as discussed further below), regardless of their gender.

## General Discussion

Across a pre-registered experiment, a replication of that experiment, and analysis of data from a public weblog, we examined the reputational costs for a social science researcher whose single study failed to replicate, and whether those costs are greater when that researcher is a woman versus a man. Results indicated a sweeping negative reaction to the researcher with the failed replication, among both the general public (i.e., adult workers from Amazon’s Mechanical Turk) and among college undergraduates; the social scientist was viewed as less competent, less likable, as having less integrity, and their entire body of work was called into question. Furthermore, both students and the general public expressed a decreased desire to interact with the researcher in the future in ways that have potential downstream negative repercussions for their career (e.g., inviting the researcher for a workplace consultation, applying to work in the researcher’s lab) when their original finding failed to replicate. Our results did not support the prediction that if the researcher was a woman, she would be more harshly penalized than if that same researcher was presented as a man. This null finding held for both public and college student perceivers. Nor did our results, based on analysis of data gleaned from a psychologist’s public weblog, find that gender of the researcher factored into the ranking of psychological scientists’ replicability status.

### Theoretical and Practical Implications

The current findings have important implications for research on the impact of the reproducibility movement on perceptions of psychologists and other social scientists, as well as people’s overall perceived value of these fields as a result of failed replications. Previous research has shown that learning about psychology’s “replication crisis” not only decreases public trust of psychological research (Anvari and Lakens, 2018), but that, perhaps because people do not differentiate among the many reasons for failed replications (many of which are not nefarious; Chopik et al., 2018), restoring public trust in psychology is an exceedingly difficult task (Wingen et al., 2020). Our experiments focused instead on failed replications of a *single* finding from an individual researcher. Consistent with prior research that also examined reputational consequences for individual social scientists with a failed study replication (Ebersole et al., 2016; Hendriks et al., 2020), our findings revealed that a failed replication broadly decreases perceptions of a researcher’s competence and scientific integrity, across their entire body of work (rather than just the particular work targeted for replication). Furthermore, our findings uniquely demonstrated consequences for perceived likability and behavioral intentions to interact with the researcher or engage with their work in the future. For example, our public sample reported being less likely to attend a public lecture by the researcher and less likely to invite them to their workplace as a consultant when their work failed to replicate than when it successfully replicated. Similarly, college students indicated that they would be less likely to take a class from or to join the research team of a researcher with a failed replication. Thus, our findings conceptually replicate and extend past findings beyond attitudinal consequences to include behavioral intentions toward researchers when findings from a single study of theirs fail to replicate.

We did not, however, find support for our gender-replication hypothesis, which was based on past and recent evidence of gender disparities in psychology and other social sciences, as well as in STEM, and based on some scholars’ characterization of academia as a masculinity contest culture (Kaeppel et al., 2020). In the present studies, we did not observe that women fared worse than men for a failed replication. Interpreting null results is always a cautious endeavor. It is clear that in both experiments, for example, some people did not pay attention to the gender of the researcher (indicated by failing the manipulation checks), but excluding those participants did not change the fact that, across a public and a college sample, participants did not evaluate the researcher differently as a function of our gender manipulation. On the one hand, this may hold promise that negative stereotypes of women researchers are fading, or that they are at least less prevalent in psychology, which is perceived to be a highly feminine field of study (Boysen et al., 2021). In this way, our findings are consistent with recent evidence demonstrating greater gender equity in the social sciences (Williams and Ceci, 2015; Bernstein et al., 2022), and this is a welcome change. On the other hand, women in psychology continue to be underrepresented in positions of leadership and influence (Gruber et al., 2021), and are less likely than men to be invited to share their work (Johnson et al., 2017; Nittrouer et al., 2018), to be cited (Brown and Goh, 2016), or to be featured prominently in graduate syllabi (Skitka et al., 2021). The fact that these disparities remain underscores the need for continued research attention to this matter and evidence-based policy changes in academia.

Our findings have practical implications for social scientists with regard to concerns about the reproducibility movement. For example, although some research suggests that researchers overestimate the personal reputational costs of failed replications (Fetterman and Sassenberg, 2015), our findings make it clear that those serious costs do exist. In the eyes of both the general public and college students, a single failed replication tarnished the researcher’s reputation and the esteem with which their work was held, and it led to more negative behavioral intentions toward the researcher. All of these outcomes may come with serious downstream career consequences, as academic researchers must demonstrate their ability to recruit students for their labs and to market their ideas to the public (e.g., broader impacts in grant submissions). Social scientists with “gatekeeper” roles, such as on academic search committees, tenure and promotion committees, and other merit review boards, should consider whether a single failed replication warrants the dismissal of one’s entire body of work versus constituting part of an effective scientific self-correcting process.

In addition, our findings echo concerns raised about the antagonistic culture surrounding the reproducibility movement. Network analyses reveal that there are two distinct clusters of literatures that have emerged from the “crisis”: one centering “open science” and the other centering “reproducibility” (Murphy et al., 2020). Analyses furthermore reveal that the open science literature is associated with more communal and prosocial descriptive language than the reproducibility literature, and women have greater representation in the open science literature than in the reproducibility literature. These findings suggest that the replication movement need not be a masculinity contest culture, where showing signs of weakness is proscribed (Berdahl et al., 2018). In light of the robust effects we observed with regard to the impact of a single failed replication on perceptions of the researcher, websites or other media that serve to raise doubts about individual researchers’ scientific integrity may contribute—even if unintentionally—to a masculine contest culture, where doubts are viewed as weak, as illustrated by items on the validated measure of masculinity contest culture (e.g., “In my work environment, admitting you do not know the answer looks weak”; Glick et al., 2018). Although we did not manipulate the researcher’s reactions to the failed replication in our experiments, participants perceived those researchers as less competent and knowledgeable, effectively admitting they did not have the “right” answers. Rather than contribute to a toxic academic culture, the findings of Murphy et al. (2020) provide reason for optimism that open science practices can serve as effective tools for improving science through transparency and educating people about the self-correcting nature of the scientific enterprise.

### Limitations and Future Directions

We note several limitations of our research. First, though we demonstrated the impact of failed replications on researcher and research perceptions among both public and student samples, the strongest test of our gender-replication hypothesis would be among a sample of researchers themselves. Although we did test this hypothesis using public weblog data to see whether members of the social science community would be more likely to target women for replication attempts, we examined only one website, which is likely not representative of the discipline at large. Another website (or list of replication efforts) might yield different results with respect to researcher gender, and, in fact, the R-Index site list itself changes with some frequency. Additionally, determining whether any list of researchers identified as candidates for replication is conclusive, and determining the appropriate benchmark with which to compare such a list, are exceedingly difficult tasks. As a result, we cannot know for sure the extent to which the current archival results are accurate and/or generalize, but we do know that the R-Index website is widely promoted on social media and thus likely highly visible. Future research might utilize algorithms to scrape other websites for information about women researchers targeted by their peers in the reproducibility movement.

Relatedly, there are important individual differences and contextual factors not addressed in the present research that likely have implications for how perceivers react to failed replications. For example, one would expect reactions to be more gendered to the extent that perceivers have implicitly or explicitly sexist views, or do not believe that women researchers face career obstacles due to gender bias (see Régner et al., 2019). In addition, given that women are penalized for displays of dominance, it might be important to examine whether they are more likely targeted by the reproducibility movement when they are especially high-status (Rudman et al., 2012).

We also included only binary categories of gender in the present research, which is a critical shortcoming of much research in psychology and other social sciences (see Tate et al., 2013; Schudson, 2021), and we did not examine the effect of researcher race or ethnicity. We expect that the marginalization related to non-binary and transgender identities may experience even greater reputational costs and potential backlash for researchers with those identities when faced with a failed replication, due to threat they elicit among perceivers (Morgenroth and Ryan, 2021). Additionally, there is reason to predict that people of color would experience a replication failure harshly (Matthew, 2016), but how this might interact with their gender identity is hard to say, given the unique stereotypes associated with intersectional identities (e.g., Ghavami and Peplau, 2013; Rosette et al., 2016). These are important avenues for future research.

Another possible limitation of our experiments concerns the topic we chose for our experimental stimuli. The embodied cognition literature has been a target of criticism, and researchers have recommended more studies from that literature follow open science best practices (Zwaan, 2021). Although, in the present studies, participants’ familiarity with prior studies of the facial feedback hypothesis did not alter results, future research on perceptions of failed replications might vary the research topic in the interest of generalizability.

Finally, future research should examine how a researcher’s reaction to a replication outcome (e.g., *via* social media or the popular press) is perceived by others. Fetterman and Sassenberg (2015) found that researchers perceived wrongfulness admission to benefit other researchers with regard to suspicion about their other work besides their finding that failed to replicate, but they did not perceive those same benefits of admitting wrongfulness for themselves. Such findings open the door for future studies to understand how a researcher can bounce back in others’ eyes after a failed replication that was not due to scientific misconduct or QRPs but instead was part of the normal self-correcting process that is scientific inquiry. Rather than putting the onus on individual researchers to deflect harsh criticism like that behind the quote with which we opened this paper, we suggest that the culture must change, especially if we are to address identity-based disparities that exist within it (see Moss-Racusin et al., 2021).

## Conclusion

The participation of women in academic social science has improved immensely over the past several decades (e.g., Ginther and Kahn, 2014; National Center for Science and Engineering Statistics Survey of Earned Doctorates (NCSES), 2021). However, the increase in women’s representation is tempered by the fact that far fewer women than men occupy positions of influence (as full professors) and power (as university leaders; e.g., Reis and Grady, 2018). As merit reviews and promotion standards at research universities often depend heavily on publications, citations, and evaluation by peers as primary indices of “impact” (Gutiérrez y Muhs et al., 2012), it is important to examine how a single social science research study that fails to replicate (versus successfully replicates) shapes opinions about and behavioral intentions toward the researcher and their work. Given the often inconclusive nature of replication studies (Etz and Vandekerckhove, 2016), our findings raise the question of whether people make broader and more negative attributions about the researcher and their entire body of work than a single failed replication warrants. In addition, given women’s underrepresentation at the highest ranks in academia (even in fields with relative gender parity overall), it is imperative to understand whether women-identified social scientists might be especially at risk for backlash when their research fails to replicate. Our work is an important first step toward answering these questions.

## Data Availability Statement

The raw data supporting the conclusions of this article will be made available by the authors, without undue reservation.

## Ethics Statement

The studies involving human participants were reviewed and approved by Indiana University. The patients/participants provided their written informed consent to participate in this study.

## Author Contributions

LA-N, CM-R, JS, TV, and PG contributed to the conceptualization and design of the studies. CM-R pre-registered the experiments. CS created experimental stimuli, tables, figures, and references. LA-N collected the data, performed the analyses, and wrote the first draft of the manuscript. All authors contributed to the article and approved the submitted version.

## Conflict of Interest

The authors declare that the research was conducted in the absence of any commercial or financial relationships that could be construed as a potential conflict of interest.

## Publisher’s Note

All claims expressed in this article are solely those of the authors and do not necessarily represent those of their affiliated organizations, or those of the publisher, the editors and the reviewers. Any product that may be evaluated in this article, or claim that may be made by its manufacturer, is not guaranteed or endorsed by the publisher.

## Footnotes

^1^In an earlier pre-registered study that yielded virtually identical results as the studies reported in this paper (for complete access to materials and data, see https://osf.io/vq2as/?view_only=354d1ad17bfa433092f0f6f5a2724265), we did not make researcher gender salient in this way. In Studies 1 and 2 of this paper we attempted to strengthen our original researcher gender manipulation through repeated reference to the researcher’s full name. Furthermore, in our initial pre-registered study, the fictitious articles containing the manipulations did not make mention of the researcher’s reactions to the failed replication of their work. In Studies 1 and 2 of this paper, we attempted to create a context (through researcher quotes in the stimulus articles) that suggested the researcher was overly self-confident (i.e., a context in which women might be more likely to be penalized). Despite this change, our findings were consistent across experiments.

^2^In addition to these critical measures, for which we had pre-registered hypotheses, we asked participants to respond to exploratory open-ended and closed-ended questions regarding their perceptions of how the researcher should feel and react to the replication outcome as well as how they personally felt upon learning about the outcome. These measures and data are available on the registration website for interested readers.

^3^ https://replicationindex.com/

^4^ https://replicationindex.com/2021/01/19/personalized-p-values/
